# Highly Selective Wet Etching of Ge Over Ge_1–_ *_x_* Sn_*x*_ Enabling Fully Released Group IV Microstructures

**DOI:** 10.1021/acsomega.6c04332

**Published:** 2026-08-17

**Authors:** Sabur Ayinde, Maksym Myronov

**Affiliations:** Physics Department, 2707 The University of Warwick, Gibbet Hill Road, Coventry CV4 7AL, U.K.

## Abstract

Selective etching between closely related group-IV semiconductors is a critical yet challenging requirement for micro and nanofabrication of advanced electronic, photonic, and thermoelectric devices. Isolating Ge_1–*x*_Sn_*x*_ epilayers from underlying Ge buffers is hindered by their similar chemical behavior and by the mechanical coupling imposed by heteroepitaxial growth on silicon. Here, we report a highly selective isotropic wet-etch process that enables the controlled removal of Ge relative to Ge_1–*x*_Sn_*x*_, facilitating the fabrication of fully released Ge_1–*x*_Sn_*x*_ microstructures. Using an ammonia-peroxide-water etchant, we demonstrate a strong composition dependence of the etch rate, with pure Ge etching at ∼150 nm/min while Ge_1–*x*_Sn_*x*_ containing ≥6.8% Sn exhibits etch rates below 17 nm/min, yielding selectivity up to ∼1:30. For lower Sn contents, selective protection via aligned lithography enables robust undercutting across the full compositional range studied. Integration with anisotropic Si etching using KOH or TMAH allows complete release of the Ge_1–*x*_Sn_*x*_ layer from the substrate. Raman spectroscopy confirms a substantial release of the initial compressive strain following removal of Ge buffer layer, with the corresponding strain relaxation estimated to be ε ≈ 0.5–0.55%. Complementary AFM analysis reveals the disappearance of cross-hatch morphology after complete suspension while maintaining a low root-mean-square surface roughness around 1 nm, confirming that the release process preserves excellent surface quality. This scalable and lithography-compatible micromachining strategy provides a robust route for selective material removal in Ge_1–*x*_Sn_*x*_/Ge/Si heterostructures, enabling the fabrication of strain-free, fully suspended GeSn microstructures. Beyond strain engineering, this approach offers a versatile platform for probing the intrinsic properties of GeSn alloys without substrate-induced constraints. The demonstrated methodology therefore opens new opportunities for the integration of suspended group-IV materials in next-generation electronic, photonic, and quantum device architectures.

## Graphical abstract

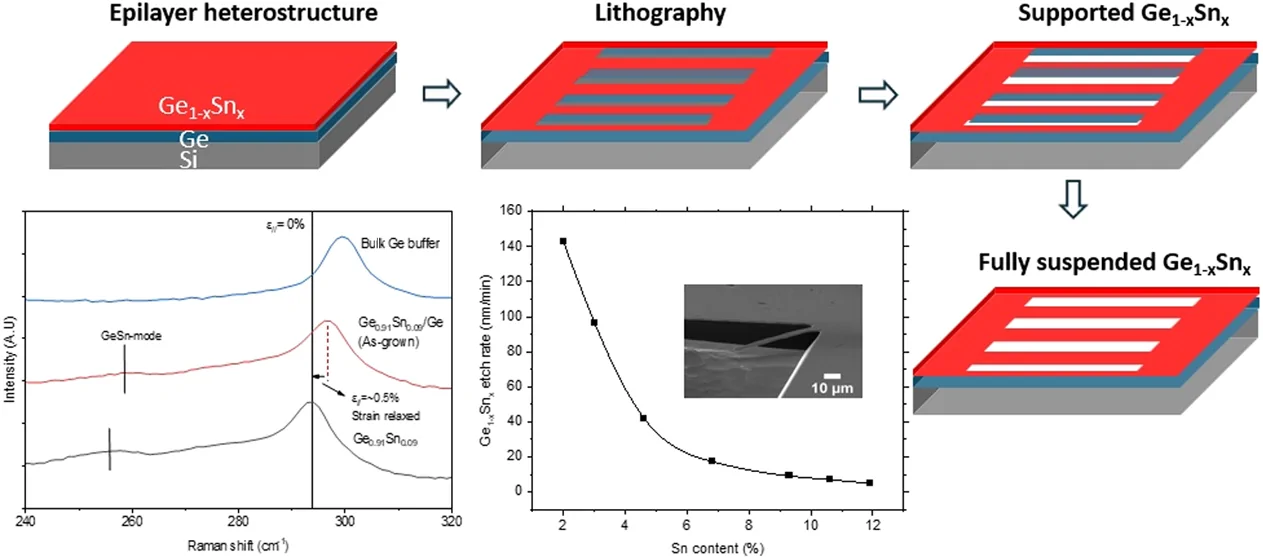

## 1 Introduction

Germanium–tin (Ge_1–*x*_Sn_*x*_) alloys have emerged as a key group-IV semiconductor material for silicon-compatible electronic,^1^ photonics,^2–7^ and energy-related technologies,^8^ owing to their tunable band structure, high carrier mobility, and compatibility with established Si processing. The composition-induced band-structure transition in Ge_1–*x*_Sn_*x*_ alloys occurs for Sn contents above ∼6.5%.^9–13^ Slight variations in Sn content leading to the indirect-to-direct bandgap transition enhance optical absorption efficiency by enabling direct electronic transitions at the Γ valley, making Ge_1–*x*_Sn_*x*_ alloys highly promising for silicon-compatible optoelectronic devices. Applications spanning infrared optoelectronics,^14–16^ lasing,^17^ high-speed electronics,^18,19^ communications,^20^ and thermoelectrics^16^ have motivated sustained interest in Ge_1–*x*_Sn_*x*_ heterostructures grown on Ge virtual substrates on silicon substrate. The versatility of GeSn alloys has been demonstrated across a wide range of device implementations. Although significant progress has been made toward achieving high crystalline quality Ge_1–*x*_Sn_*x*_ alloys via strain relaxation strategies,^21–24^ heteroepitaxial systems inherently introduce complications. The underlying buffer/substrate layer introduces residual strain,^25^ parasitic electrical pathways,^26^ optical interference in photonic devices,^27,28^ and thermal coupling,^29^ thereby complicating both fundamental characterization and device optimization.

Selective removal of sacrificial layers is a cornerstone of micro- and nanofabrication, enabling material isolation, stress relaxation, and the creation of free-standing architectures. In the Ge_1–*x*_Sn_*x*_/Ge system, this task is particularly challenging because of the close chemical similarity between the two materials and the mechanical dependence of strained Ge_1–*x*_Sn_*x*_ epilayers on the underlying Ge buffer.

Although dry etching remains the preferred technique for fabricating submicrometer and highly anisotropic structures, as existing approaches to Ge_1–*x*_Sn_*x*_ microstructure fabrication have largely relied on selective dry etching,^30–32^ complex postgrowth processing,^33^ or selective growth on patterned Si(001). These methods often lead to process complexity, introduce increased defect densities,^34^ and limit achievable Sn incorporation.^35^ Selective epitaxial growth combined with partial undercutting generally results in residual support pillars or requires complex fabrication routes.^32,33^ Over the years, several approaches have been proposed for strained laser devices, such as suspended microbridges,^36^ microdisk cavities,^37^ and recently, group IV semiconductors on insulators^38^ with stressors for tensile strain injection. The common goal of these techniques is to remove compressive strain and misfit defects, which disintegrate the optical gain properties of the active material.^39^ However, thermal dissipation, complex fabrication process, and difficulty in demonstrating electrically driven devices remain the major challenges with these approaches.

By completely undercutting the sacrificial layer beneath the active layer, the compressive strain can be released. Reports of fully released, free-standing Ge_1–*x*_Sn_*x*_ structures realized via simple and scalable wet-chemical processes remain limited. Unlike dry etching, which relies on plasma-assisted material removal that may introduce ion-induced surface damage, the selective wet etching process provides a simple and highly selective route for strain engineering and structural release of Ge_1–*x*_Sn_*x*_ layers. Also, wet chemical etching offers inherent advantages in scalability, throughput, and compatibility with established semiconductor manufacturing. Ammonia–peroxide mixtures have long been employed in silicon processing and have been explored for etching Ge-based materials. Prior studies have indicated that etch selectivity between Ge and Ge_1–*x*_Sn_*x*_ increases with Sn content,^32,40^ but achieving robust selectivity across a broad compositional range-especially at low Sn concentrations, remain non- trivial.

In this work, we demonstrate a composition-dependent selective wet-etch strategy that enables controlled removal of Ge relative to Ge_1–*x*_Sn_*x*_ in Ge_1–*x*_Sn_*x*_/Ge/Si heterostructures. By combining isotropic etching in an ammonia–peroxide mixture with anisotropic silicon micromachining and lithographic protection where required, we achieve full release of Ge_1–*x*_Sn_*x*_ microstructures without resorting to complex fabrication process or postgrowth processing. We systematically quantify the etch-rate dependence on Sn concentration, establish practical selectivity limits, and demonstrate the fabrication of fully suspended Ge_1–*x*_Sn_*x*_ cantilevers, beams, microwires, and membranes.

Structural and compositional properties of the Ge_1–*x*_Sn_*x*_ epilayers are characterized using high-resolution X-ray diffraction (HRXRD) and transmission electron microscopy (TEM), where interference fringes and reciprocal space maps enabled extraction of Sn composition, strain, relaxation, and crystallinity, while cross-sectional TEM provided direct thickness measurements and confirmed high-quality heterointerfaces. Raman spectroscopy confirmed relaxation of the initial compressive strain following removal of the Ge buffer, while atomic force microscopy (AFM) was used to evaluate surface quality and the effects of suspension on strain relaxation. The demonstrated methodology establishes a versatile platform for strain engineering, offers a general route for selective material removal in group-IV heterostructures, and underpins future studies of suspended GeSn-based materials and devices.

## 2 Results and Discussion

### 2.1 Isotropic/Anisotropic Etching

High-fidelity pattern material selectivity was achieved using wet chemical etching via lithography, ensuring dimensional fidelity and precise structural feature alignment. A comparison of the most commonly used wet etchants, including their etching characteristics, relative etch rates, as reported in literature, is summarized in Table 1. The compatibility of the etching process with different resist chemistries was evaluated in this work using both positive and negative photoresists. Ge_1–*x*_Sn_*x*_/Ge/Si heterostructure samples were cleaved into ∼1 cm^2^ pieces and cleaned by sequential ultrasonic baths in acetone and isopropanol (5 min each), rinsed in deionized water under nitrogen, and eventually dehydrated at 90 °C for 1 min. Photoresists were spin-coated and exposed using a micropattern generator. It was realized that both photoresists were compatible with subsequent etching steps.

**Table 1:** Summary of Isotropic and Anisotropic Etching of Silicon

etching chemicals	reactions	etch rate	references
HF & HNO_3_	isotropic etching with smooth surface as HF dissolves the SiO_2_ formed from oxidation of Nitric acid	moderate to high	^41,42^
KOH	anisotropic etching due to orientation dependence. depends on concentration and temperature	slow to high	^43,44^
TMAH	anisotropic controlled etching with slow etch rate	slow to moderate	^45,46^
NaOH	anisotropic etching, leaving smooth surface with moderate selectivity	moderate	^47–49^

The wafer sample for positive-tone lithography, S1813 was spin-coated at 4000 rpm for 30s to obtain a thickness of ∼1.6 μm (Figure 1b). The micropattern generator exposes the pattern design as schematically illustrated in Figure 1c, with inverted mask polarity to accommodate the positive-tone resist. Development was carried out in AZ 326 MIF for 30s (Figure 1d), followed by deionized water rinsing and nitrogen drying.

**Figure 1:**
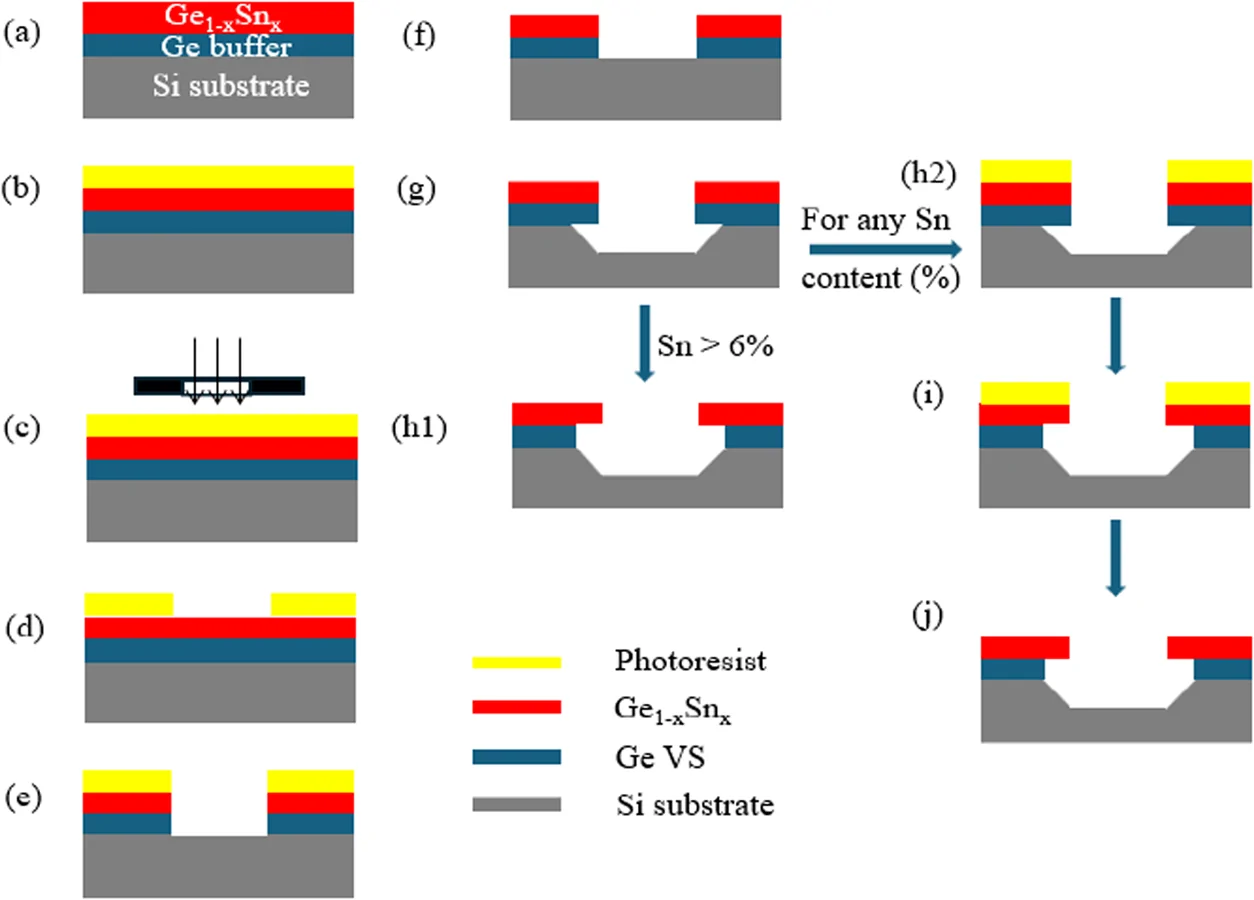
Schematic illustrating the key steps involved in achieving a fully suspended single layer via selective etching. (a) As-grown Ge_1–*x*_Sn_*x*_/Ge/Si heterostructure. (b) Spin-coating of photoresist on the sample surface. (c) UV lithography exposure using micropattern generator. (d) Development of the exposed sample in AZ326MIF developer. (e) Isotropic chemical wet etching using an ammonia-peroxide consisting of NH_4_OH, H_2_O_2_, and deionized water (H_2_O) in the ratio 2.5:5:80. (f) Removal of photoresist layer by acetone wash. (g) Anisotropic wet etching of the Si substrate performed in either 20% KOH or 25% TMAH solution at 90 °C in water bath at 300 rpm. (h1) Selective wet etching using the ammonia peroxide mixture for samples with Sn concentration greater than 6%. (h2) Aligned lithography step, to protect the top layer with photoresist for samples with any Sn concentration. (i) Selective removal of the Ge layer relative to the Ge_1–*x*_Sn_*x*_ layer. (j) Removal of photoresist by acetone wash yielding the completed suspended structure.

### 2.2 Dependence of Ge_1–*x*_Sn_*x*_ Etch Rate on Sn Content

The etch selectivity of Ge over Ge_1–*x*_Sn_*x*_ was evaluated after releasing the Ge_1–*x*_Sn_*x*_/Ge mesa structures from the Si substrate realized by anisotropic wet etching, resulting in suspended mesas (Figure 1g). Two complementary approaches were employed to probe the etch selectivity of both Ge and Ge_1–*x*_Sn_*x*_, as schematically illustrated in Figure 1h.

In method 1 (Figure 1h1), the suspended Ge_1–*x*_Sn_*x*_/Ge mesa microstructures were directly immersed in an ammonia-peroxide mixture. This method was applied to samples with Sn concentrations exceeding 6%, for which the Ge_1–*x*_Sn_*x*_ epilayers exhibited substantially enhanced resistance to the etchant relative to that of Ge, enabling selective removal of the underlying Ge buffer layer without additional masking.

In method 2 (Figure 1h2), a micromachining-based approach was used to widen the selectivity studies across the full compositional range. A photoresist layer was spin-coated onto the suspended mesa structures, followed by a second lithography step to repattern the original design with precise overlay alignment. This process protected the Ge_1–*x*_Sn_*x*_ epilayer during subsequent etching, independent of Sn concentration. The samples were then etched in the ammonia–peroxide mixture for approximate durations corresponding to complete removal of the Ge layer (etch rate at 150 nm/min). As observed from the nonlinear dependence on Sn content (Figure 3), the slight reduction in etch rate between 0 and 2% Sn content can be attributed to the onset of localized surface inhibition arising from the formation of isolated SnO_2_ species, which are less soluble than GeO_2_, rather than significant surface passivation. In contrast, the sharp decrease in etch rate beyond 2% Sn indicates a transition to a dissolution-limited regime, associated with the development of a quasi-continuous, more stable, passivating SnO_2_-rich surface layer. The observed threshold highlights the strong sensitivity of wet-etching kinetics to small changes in Sn composition and provides insight into the origin of the high Ge-over- Ge_1–*x*_Sn_*x*_ selectivity achieved in this work. Using the wet etch approach, fully suspended, relaxed Ge_1–*x*_Sn_*x*_ mesa structures were reproducibly obtained for all compositions studied.

The fabrication approach described above enables fully suspended single-layer microstructures, including cantilevers, microwires, and cross-beams. These geometries are well suited for applications such as sensing devices, mechanical resonators, on-chip optical cavities, and thermoelectric microgenerators. The method is inherently limited to discrete structures defined by top-down patterning.

To enable continuous, large-area suspended membranes, a bottom-up fabrication strategy was developed. Figure 2 schematically illustrates the key steps of the approach. In contrast to the top-down processes shown in Figure 1, this approach enables the formation of continuous, single-layer large-area Ge_1–*x*_Sn_*x*_ thereby extending the platform beyond localized microstructures toward membrane-based device architectures.

**Figure 2:**
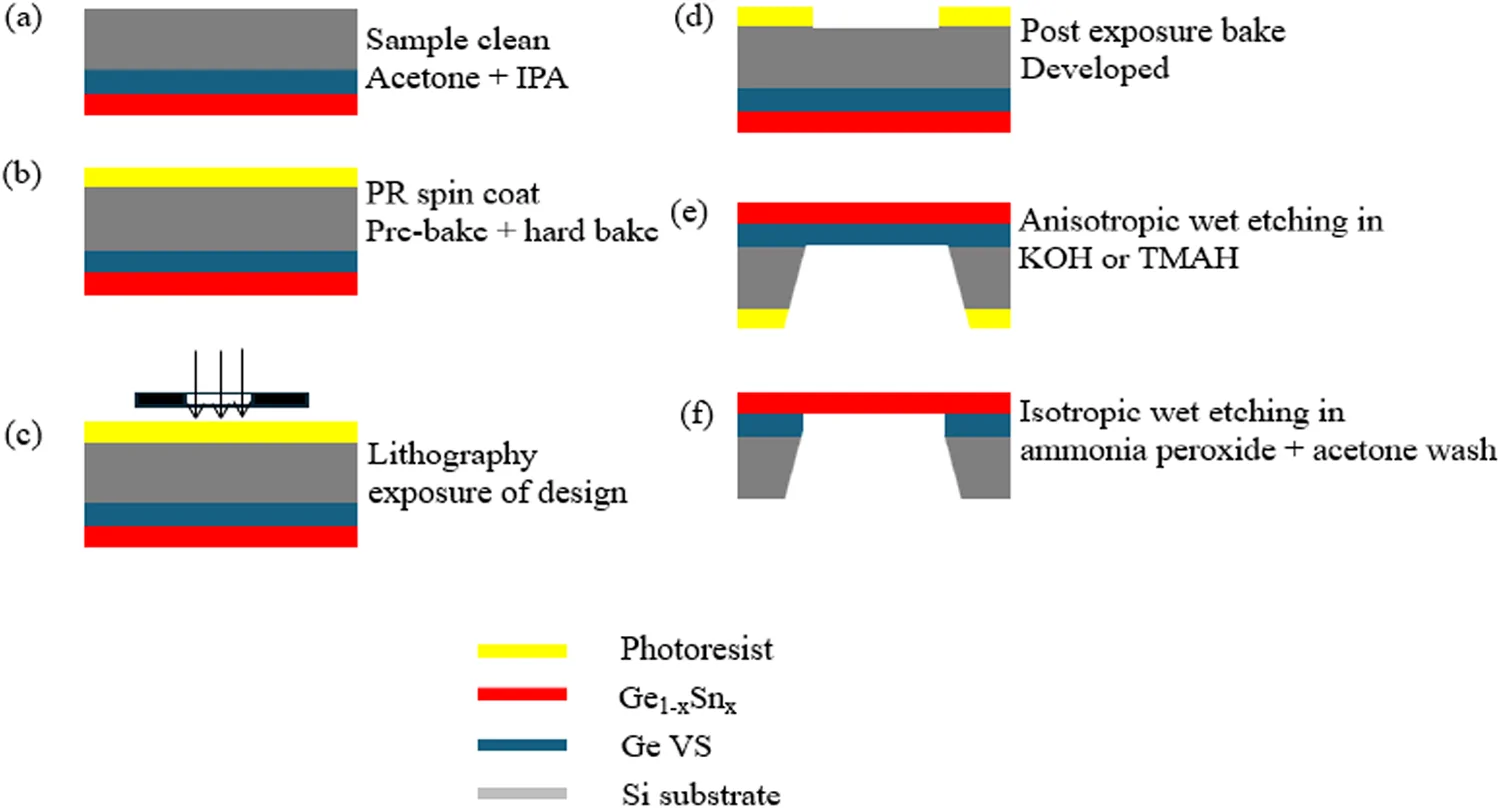
Schematic illustration of key fabrication steps for the fabrication of membrane from a grown Ge_1–*x*_Sn_*x*_/Ge/Si heterostructure. (a) Grown wafer sample cleaned in an acetone wash followed by isopropyl alcohol (IPA). (b) Photoresist coated on the backside of the sample (Si surface). (c) Design pattern transferred using laser writer. (d) Post-exposure baking is performed, followed by development. (e) Anisotropic chemical wet etching of the Si substrate. (f) Isotropic chemical wet etching of the underlying Ge layer fully suspends the single-layer membrane.

Across all fabricated microstructures, Ge_1–*x*_Sn_*x*_ alloys exhibited a significant reduction in etch rate with increasing Sn content under identical ammonia–peroxide etching conditions. At a Sn concentration of 11.9%, the measured etch rate decreased to 4.8 nm min^–1^, compared with ∼150 nm min^–1^ for pure Ge (Figure 3). The etch rate was found to decrease exponentially with increasing Sn concentration, as experimentally verified in this study. Extrapolation of this trend indicates that Ge_1–*x*_Sn_*x*_ alloys with Sn contents approaching ∼13% become effectively inert to the ammonia–peroxide mixture at the composition employed.

**Figure 3:**
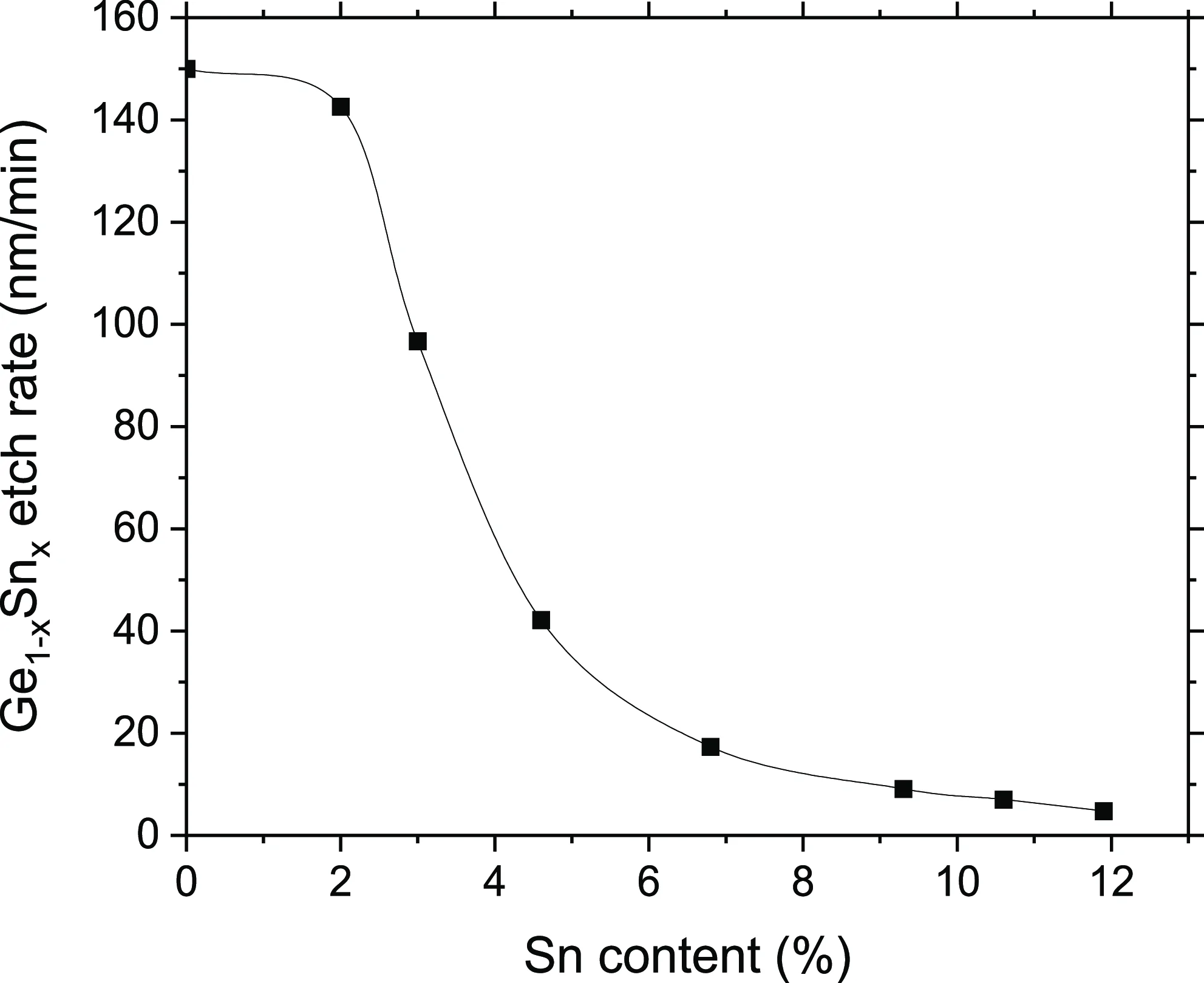
Etch rate as a function of Sn content (%) showing an exponential decrease in etch rate with increasing Sn content in Ge_1–*x*_Sn_*x*_.

These results demonstrate that increasing Sn incorporation substantially enhances the chemical resistance of Ge_1–*x*_Sn_*x*_, leading to a marked improvement in etch selectivity relative to Ge and enabling reliable selective removal of Ge layers from the Ge_1–*x*_Sn_*x*_/Ge heterostructures.

### 2.3 Fabricated Microstructures

Scanning electron microscopy images and atomic force microscopy (AFM) of the surface morphology of the fabricated microstructures are presented in Figure 4. Figure 4a represents the SEM image of as-grown Ge_1–*x*_Sn_*x*_/Ge mesa microwires on a Si substrate. Following the initial anisotropic Si undercutting, suspended Ge_1–*x*_Sn_*x*_/Ge-based microwires with widths ranging from 10 to 50 μm and lengths up to ∼1000 μm were obtained (Figure 4b,c). During KOH etching, residual surface deposits were observed on the etched structures, consistent with previous reports,^50^ whereas such residues were not observed for TMAH-based etching. Although these residues were readily removed by RCA-1 clean at (≤10 s), resulting in contaminant-free microstructures.

**Figure 4:**
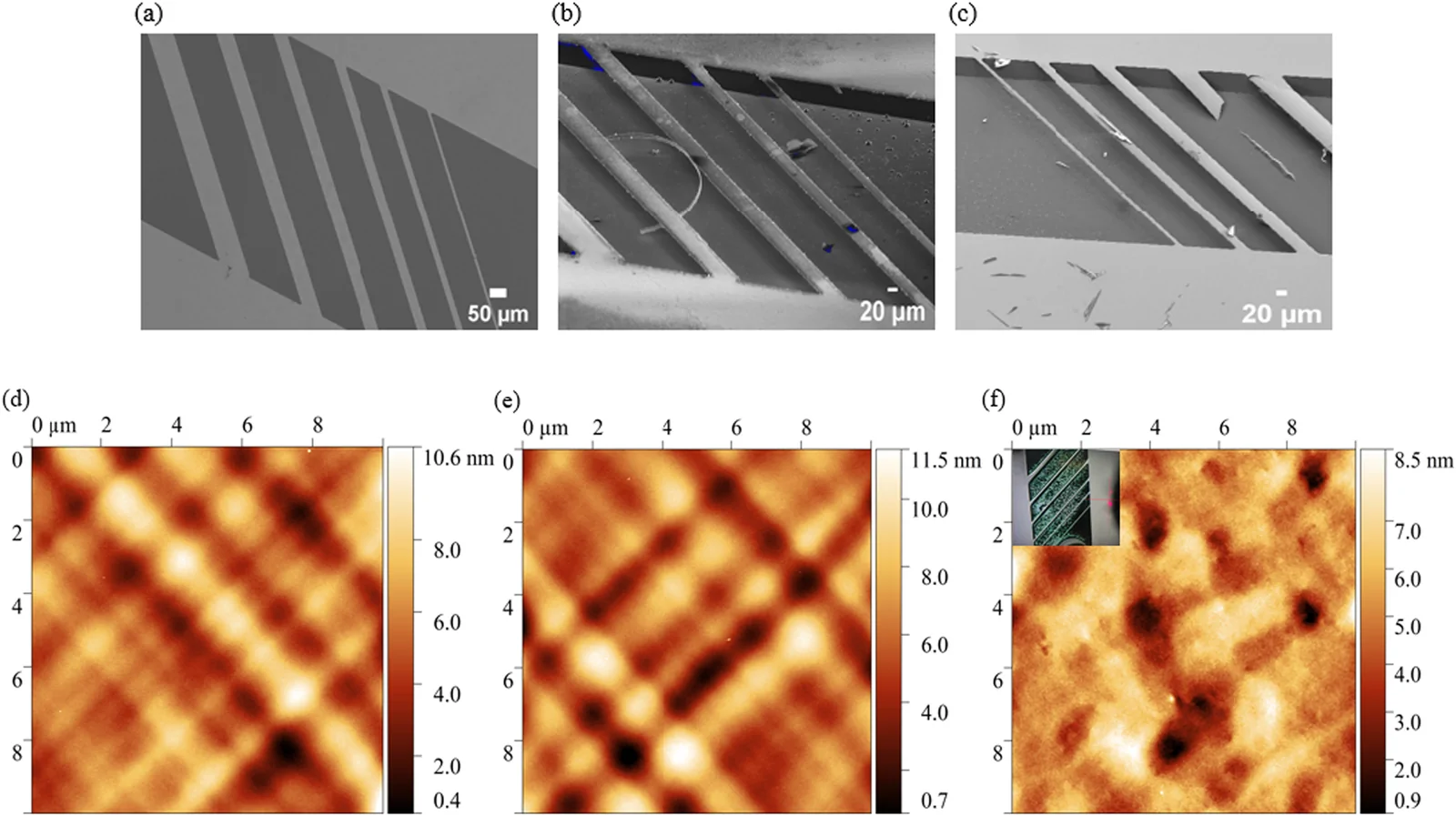
SEM imaging and AFM surface morphology of microfabricated structures. (a) Formation of unsuspended Ge_1–*x*_Sn_*x*_/Ge microwires through isotropic wet etching in an ammonia–peroxide mixture at room temperature. (b) Suspended Ge_1–*x*_Sn_*x*_/Ge mesa structures obtained by anisotropic wet chemical etching of Si in KOH at 90 °C (etch rate of 0.6 μm/min), producing microwires 10–50 μm wide and up to 1000 μm long. (c) Selective removal of the Ge layer relative to Ge_1–*x*_Sn_*x*_ achieved either by direct immersion in the ammonia-peroxide solution for Sn concentration >6% (high etch selectivity) or by lithography alignment to protect the top layer prior to timed etching of the underlying Ge. AFM surface topography of (d) the as-grown Ge_1–*x*_Sn_*x*_/Ge/Si heterostructure, (e) the suspended Ge_1–*x*_Sn_*x*_/Ge structure, and (f) the fully suspended Ge_1–*x*_Sn_*x*_ layer, with an inset on the top left corner showing screenshot of when Bruker AFM is being used for the measurements. The AFM images were acquired over 10 × 10 μm^2^ scan areas with scan axes rotated by 45° relative to the 110 crystallographic directions. The reported RMS roughness values represent averages from three spatially separated scan regions for each sample. Cross-hatch features are evident in the unsuspended Ge_1–*x*_Sn_*x*_/Ge/Si and suspended Ge_1–*x*_Sn_*x*_/Ge samples, whereas these features disappear after complete suspension of the Ge_1–*x*_Sn_*x*_ layer, indicating strain relaxation while maintaining excellent surface quality.

Figure 4b corresponds to a suspended Ge_1–*x*_Sn_*x*_/Ge mesa, while Figure 4c shows fully suspended microwires realized by isotropic undercutting of the underlying Ge buffer layer. Complete suspension was realized via selective removal using either of the two approaches discussed above and was reproducible for both strained and relaxed Ge_1–*x*_Sn_*x*_ epilayers with thicknesses ranging from ∼ 30 to 500 nm.

AFM measurements acquired over 10 × 10 μm^2^ scan areas (as in Figure 4d–f) demonstrate that the selective release process preserves the surface quality of the Ge_1–*x*_Sn_*x*_ layer. The root-mean-square (RMS) surface roughness values were determined from three spatially separated scan regions for each sample and are reported as the mean ± standard deviation. The as-grown Ge_1–*x*_Sn_*x*_/Ge/Si heterostructure exhibited an RMS roughness of 1.6 ± 0.1 nm (Figure 4d), while the suspended Ge_1–*x*_Sn_*x*_/Ge and fully suspended Ge_1–*x*_Sn_*x*_ structures showed RMS roughness of 1.7 ± 0.15 nm (Figure 4e), and 1.2 ± 0.2 nm (Figure 4f), respectively. Figure 4d,e revealed the presence of cross-hatch surface morphology in both the unsuspended Ge_1–*x*_Sn_*x*_/Ge/Si heterostructure and the partially suspended Ge_1–*x*_Sn_*x*_/Ge sample, consistent with strain relaxation through the underlying misfit dislocation network. In contrast, no discernible cross-hatch features were observed in the fully suspended Ge_1–*x*_Sn_*x*_ layer as in Figure 4f. This suppression of the cross-hatch morphology following complete release is consistent with strain redistribution within the suspended structure. A pronounced dependence of structural integrity on lateral dimensions was observed. While microstructures with widths between 10 and 30 μm consistently remained intact, wider structures (≥40 μm) frequently exhibited cracking or fracture during release (Figure 4c). This behavior was observed across multiple compositions and is exemplified by the ∼58 nm thick strained Ge_0.91_Sn_0.09_ microwires, indicating that mechanical stability becomes a limiting factor for large-area single-layer of suspended Ge_1–*x*_Sn_*x*_ microstructures. Figure 5 presents optical and SEM images of fabricated single-layer suspended Ge_1–*x*_Sn_*x*_ structures realized using both top-down and bottom-up fabrication strategies. Using the top-down etching process described in Figure 1, suspended cantilevers (Figure 5a,e), crossbeams (Figure 5b,f), and microwires (Figure 5c,g) were fabricated with well-defined design geometries and lateral confinement printed by lithographic patterning.

**Figure 5:**
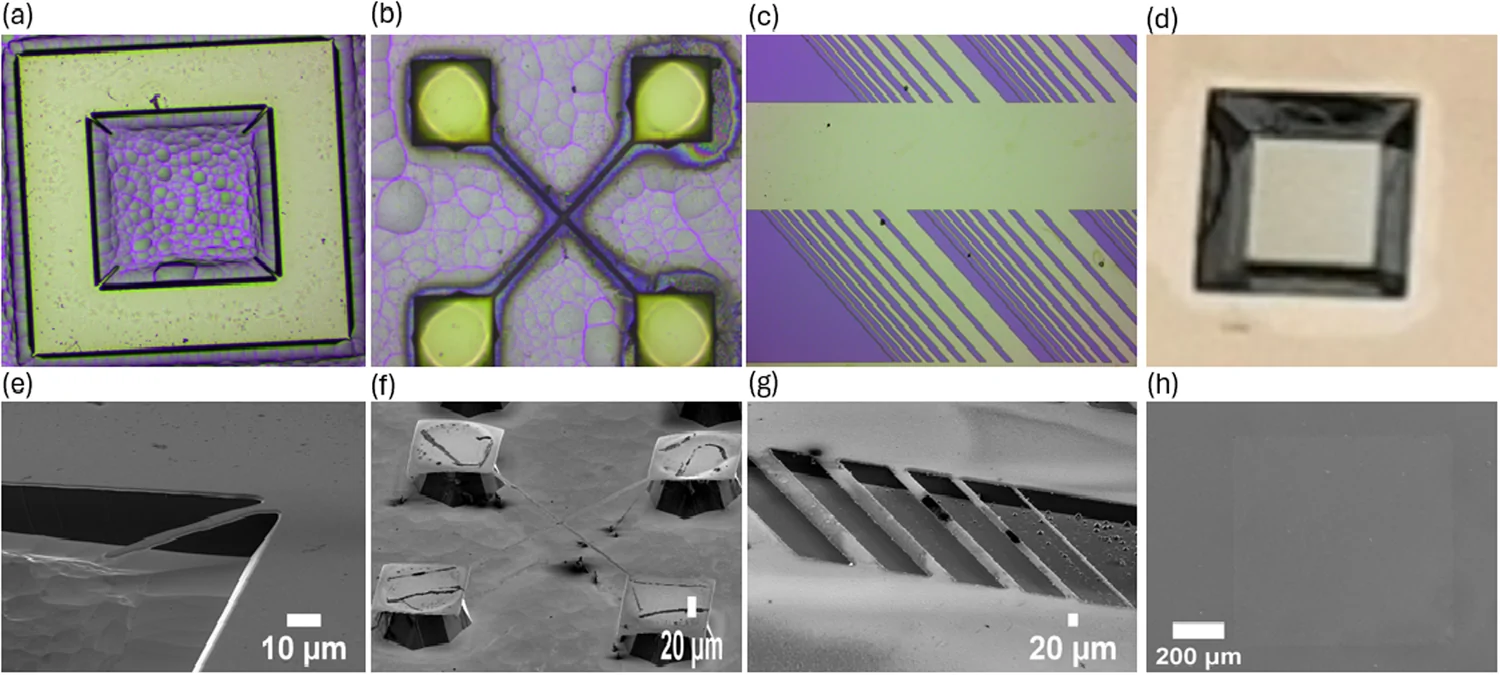
Optical and scanning electron microscopy (SEM) images of microfabricated suspended single-layer Ge_1–*x*_Sn_*x*_ structures. Optical micrographs of (a) diagonally aligned cantilevers in the design layout, (b) crossbeam structures, (c) microwires with widths of 10–50 μm and lengths up to 1000 μm, and (d) a suspended membrane. Corresponding SEM images of the same structures: (e) cantilever with dimensions of 2.5 × 30 μm, (f) crossbeam measuring 10 × 500 μm, (g) microwire (10–50 μm wide, 1000 μm long) viewed at a 55° tilt angle, and (h) suspended membrane with lateral dimensions of 400 × 412 μm.

These discrete microstructures enable localized mechanical and optoelectronic functionalities and are particularly suitable for device concepts requiring isolated suspended elements. In contrast, Figure 5d,h shows a fully suspended, strain-free Ge_1–*x*_Sn_*x*_ membrane fabricated using the bottom-up etching as shown in Figure 2.

Ge_1–*x*_Sn_*x*_ epilayers grown on a Ge buffer layer are intrinsically under compressive strain, which can limit their performance in advanced electronics,^25^ as such parasitic limitations have recently been demonstrated in thermal transport measurements.^29^

### 2.4 Strain-Free Selective Etching of Ge_1–*x*_Sn_*x*_

Raman spectroscopy was employed to investigate the strain state of the Ge_0.91_Sn_0.09_ layer before and after suspension. Figure 6 compares the Raman spectra of the as-grown Ge_0.91_Sn_0.09_/Ge heterostructure with those obtained from the selectively etched, fully suspended Ge_0.91_Sn_0.09_ epilayer and the Ge–Ge vibrational mode of the bulk Ge buffer layer used as a reference. In the as-grown structure, the Ge_0.91_Sn_0.09_ film exhibits a Raman peak shift relative to bulk Ge, indicating the presence of compressive strain originating from lattice mismatch with the underlying Ge layer. Following selective removal of the Ge layer, the suspended ∼58 nm thick Ge_0.91_Sn_0.09_ film shows a clear shift of the Raman peak position of −∼2.18 cm^–1^, demonstrating significant strain relaxation. This behavior confirms that the micromachining and selective etching processes effectively release the compressive strain initially present in the epitaxial layer. It is noted that no observable significant change in the Raman peak line width of the fully suspended Ge_1–*x*_Sn_*x*_, following the selective release process. The preservation of the Raman line width is an indication that the etching process does not introduce measurable crystalline disorder or a form of strain inhomogeneity.

**Figure 6:**
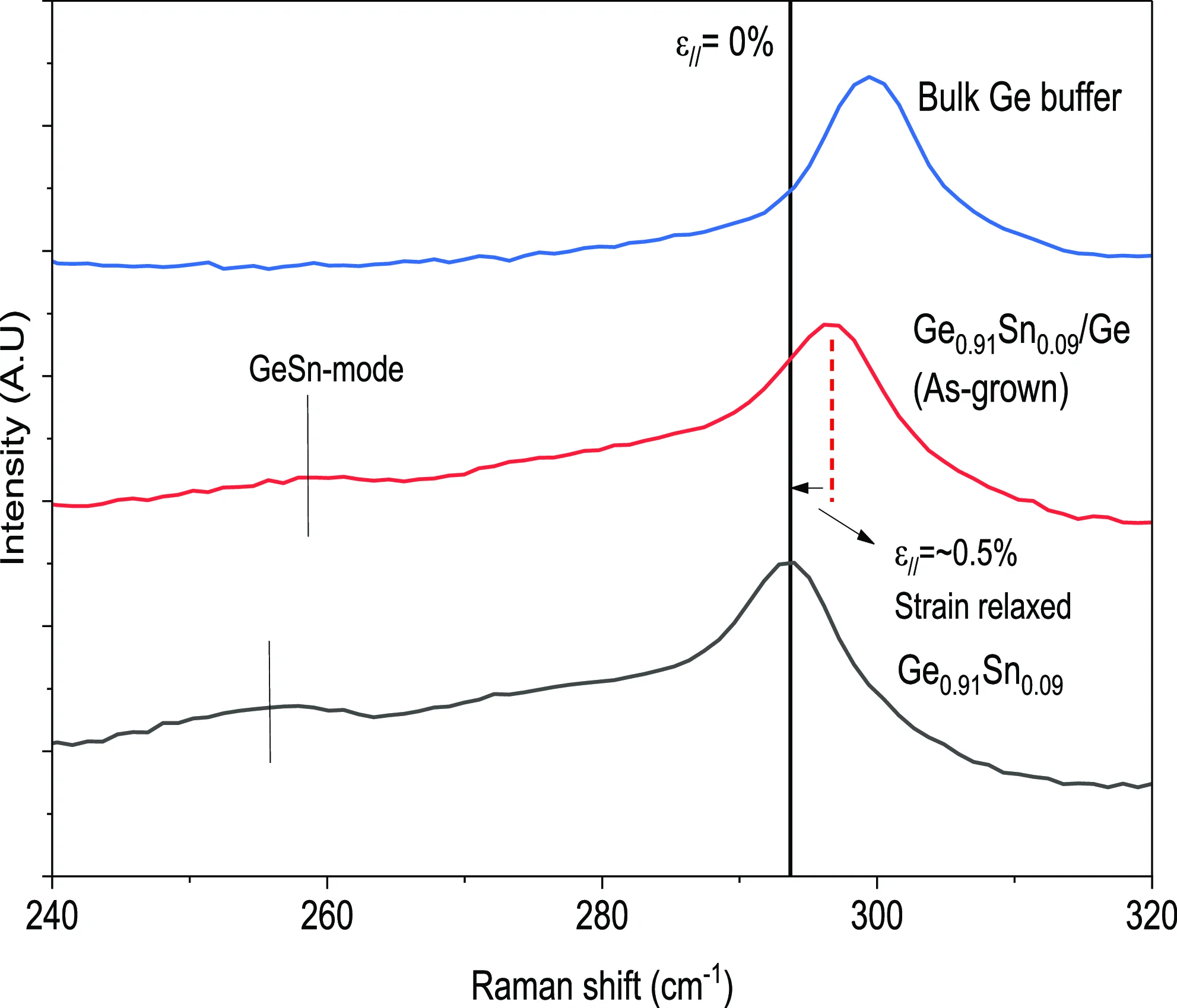
Raman spectra of the as-grown, supported Ge_0.91_Sn_0.09_/Ge heterostructure compared with a selectively etched, fully suspended Ge_0.91_Sn_0.09_ epilayer and bulk Ge reference. The comparison highlights strain relaxation in the ∼58 nm thick strained Ge_0.91_Sn_0.09_ film following removal of the underlying Ge layer.

For Ge_0.91_Sn_0.09_, the Ge–Ge longitudinal optical (LO) phonon peak was observed at 296.15 cm^–1^ for the supported Ge_0.91_Sn_0.09_/Ge heterostructure and at 293.97 cm^–1^ for the suspended Ge_1–*x*_Sn_*x*_ epilayer, corresponding to a redshift of ∼2.18 cm^–1^ upon release. Noting that the Raman shift, Δω = −2.18 cm^–1^ and considering an approximate value of 400–450 cm^–1^/strain for the phonon deformation potential coefficient for Ge_0.91_Sn_0.09_, the corresponding strain relaxation is estimated to be ε ∼ 0.5–0.55%, indicating substantial release of the initial compressive strain upon suspension.

This Raman redshift directly indicates relaxation of the compressive strain following selective removal of the sacrificial Ge layer, confirming effective decoupling of the Ge_1–*x*_Sn_*x*_ epilayer from the Ge virtual substrate. The Ge–Ge LO phonon peak of Ge_0.91_Sn_0.09_/Ge appears at a lower wavenumber than that of bulk Ge (∼299.7 cm^–1^), reflecting phonon softening due to Sn incorporation. Consistent with this behavior, the Ge–Ge LO phonon frequency systematically decreases with increasing Sn content in Ge_1–*x*_Sn_*x*_ alloys. Recent reports on strain-free GeSn^38,51^ are not only realized by complex processes, they have also primarily been demonstrated on non Si-compatible platforms, which lack reproducibility and scalability for large-scale manufacturing. The suppression of cross-hatch as observed from the surface morphology of the fully suspended Ge_1–*x*_Sn_*x*_ as in Figure 4f, agrees with the strain relaxation observed from Raman measurements (Figure 6). Importantly, the disappearance of the cross-hatch pattern occurred without significant degradation of surface quality, with the average RMS roughness around 1.16 nm.

The selective wet etching approach developed in this work offers several advantages for the fabrication of suspended Ge_1–*x*_Sn_*x*_ structures. Unlike conventional dry etching, which is primarily employed for pattern definition, the present method exploits the exceptionally high etch selectivity between Ge and Ge_1–*x*_Sn_*x*_ to selectively remove Ge sacrificial layers while preserving the Ge_1–*x*_Sn_*x*_ active layer. This enables efficient release of suspended microstructures, promotes strain relaxation, and avoids plasma-induced damage associated with ion-assisted etching processes. Furthermore, AFM and Raman analyses confirm that the release process preserves both the surface morphology and crystalline quality of the Ge_1–*x*_Sn_*x*_ layer, highlighting the potential of this approach for future Ge_1–*x*_Sn_*x*_ photonic, MEMS, and strain-engineered device applications.

## 3 Conclusion

We have demonstrated a robust and scalable wet-chemical etching strategy that enables highly selective removal of Ge relative to Ge_1–*x*_Sn_*x*_ in Ge_1–*x*_Sn_*x*_/Ge/Si heterostructures. Using an ammonia-peroxide etchant, we establish a strong composition dependence of the etch rate, exhibiting etch rates below 17 nm/min, yielding selectivity around 1:30. For lower Sn concentrations, selective protection via aligned lithography enables controlled undercutting across the full compositional range studied.

By integrating isotropic Ge_1–*x*_Sn_*x*_/Ge etching with anisotropic Si micromachining using KOH or TMAH, complete release of Ge_1–*x*_Sn_*x*_ layers from both the Ge buffer and the Si substrate was achieved, enabling the formation of fully suspended cantilevers, beams, microwires, and membranes.

Raman spectroscopy confirms the relaxation of compressive strain following removal of the Ge sacrificial layer, validating the effectiveness of the selective etching process in mechanically decoupling the underlying buffer layer from the active epilayer. This interpretation is further supported by AFM measurements, which reveal the disappearance of cross-hatch morphology with low RMS surface roughness ∼1 nm from the fully suspended Ge_1–*x*_Sn_*x*_ microstructure. These results indicate that the selective release process enables efficient strain relaxation while preserving the excellent surface quality of the microstructures.

The presented approach avoids complex postgrowth processing and is fully compatible with standard lithographic techniques, providing a scalable route for selective material isolation in Ge_1–*x*_Sn_*x*_/Ge/Si heterostructures. Raman analysis reveals a significant strain relaxation of ∼0.5% upon suspension, effective decoupling of the Ge_0.91_Sn_0.09_ epilayer from the underlying Ge layer and highlighting the potential of this method for realizing strain-free group-IV microstructures. The ability to selectively remove Ge while realizing a strain-relaxed Ge_1–*x*_Sn_*x*_ layer provides a versatile platform for investigating suspended group-IV materials. The significant strain relaxation demonstrated here further highlights the potential of this approach for enabling advanced micro- and nanoarchitectures for electronic, photonic, thermoelectric, and MEMS-based applications. Beyond Ge_1–*x*_Sn_*x*_, this work establishes general design principles for composition-dependent selective wet etching in multicomponent semiconductor systems. Future work will focus on photoluminescence characterization of the suspended Ge_1–*x*_Sn_*x*_ microstructures to further investigate the influence of strain relaxation on their optical properties and assess their suitability for photonic device applications.

## 4 Experimental Methods

### 4.1 Material Growth

Ge_1–*x*_Sn_*x*_ layers were grown by reduced-pressure chemical vapor deposition (RPCVD) in an Epsilon 2000 industrial semiconductor reactor. Growth was carried out at temperatures between 240 and 340 °C on Ge virtual substrates, which were first deposited at higher temperature on Si substrates.

### 4.2 X-ray Diffraction

Film composition and thickness were determined by high-resolution X-ray diffraction (HRXRD) through analysis of interference fringes from strained Ge_1–*x*_Sn_*x*_ layers. Symmetric and asymmetric reciprocal space maps (RSMs) were acquired for both strained and relaxed samples to evaluate Sn composition, degree of relaxation, lattice tilt, and strain state.

### 4.3 Cross-Sectional TEM

Epilayer thicknesses were determined by cross-sectional transmission electron microscopy (XTEM). Strained Ge_1–*x*_Sn_*x*_ samples with thicknesses ranging from ∼30 to 87 nm and relaxed samples with thicknesses between ∼300 and 500 nm were investigated. XTEM analysis of both strained and relaxed Ge_1–*x*_Sn_*x*_/Ge/Si heterostructures provided precise thickness measurements. High-resolution TEM further confirmed the high crystalline quality of the grown epilayers.

### 4.4 Bottom-Up Fabrication

For membrane fabrication, samples were cleaned in isopropyl alcohol prior to backside lithography. S1813 positive photoresist was spin-coated at 4000 rpm for 30 s to yield a thickness of ∼1.3 μm, followed by a prebake at 120 °C for 3 min. Backside patterning was performed using a μPG lithography system (WMIII mode, filter off, 5.0 bar pressure, 90% of 20 mW laser power, exposure energy of 1 × 1). The design was exposed without polarity inversion. Development was carried out in AZ 326 MIF for 30 s, followed by deionized water rinsing and nitrogen drying.

To protect the patterned features, the top surface of the sample was coated with an additional S1813 photoresist layer by spin coating at 4000 rpm for 30 s. The protected sample was briefly etched in an ammonia–peroxide mixture (H_2_O_2_:NH_4_OH:H_2_O = 5:2.5:80) for 30 s, followed by rinsing in deionized water and nitrogen drying. The protective photoresist layer was subsequently removed by acetone spray, and the sample was dried under nitrogen.

### 4.5 Etching Process

Deep silicon etching was performed using either 25 wt % TMAH or KOH at 90 °C in a stirred water bath (300 rpm) to ensure uniform temperature distribution. After etching, samples were gently rinsed in three successive deionized water baths and allowed to air-dry without nitrogen blow-drying to avoid mechanical damage to the suspended mesa structures.

### 4.6 Raman Spectroscopy

Raman measurements were carried out using 633 nm excitation laser with a 50× objective, corresponding to an approximate spot size of ∼1 μm, at a nonheating laser power density of ∼25 mW/m^2^.

### 4.7 Atomic Force Microscopy

The surface morphology and surface quality of etched/microfabricated structures were observed using a Bruker Icon AFM.
